# Evaluation of Milled Titanium versus Laser Sintered Co-Cr Abutments on the Marginal Misfit in Internal Implant-Abutment Connection

**DOI:** 10.3390/ma13214873

**Published:** 2020-10-30

**Authors:** Esther Gonzalo, Beatriz Vizoso, Carlos Lopez-Suarez, Pedro Diaz, Jesus Pelaez, Maria J. Suarez

**Affiliations:** Department of Conservative Dentistry and Bucofacial Prosthesis, Faculty of Odontology, University Complutense of Madrid, 28040 Madrid, Spain; esgonzal@ucm.es (E.G.); beavizoso@hotmail.com (B.V.); carlop04@ucm.es (C.L.-S.); pediaz02@ucm.es (P.D.); jpelaezr@ucm.es (J.P.)

**Keywords:** Co-Cr abutment, laser sintering, CAD/CAM milled, marginal misfit, implant-abutment interface

## Abstract

The precision of fit at the implant-abutment connection is an important criterion for the clinical success of restorations and implants. Several factors are involved among which are the abutment materials and manufacturing techniques. The aim of this study was to investigate the effect of two materials and methods of manufacturing implant abutments, milled titanium versus laser sintered Co-Cr, on the marginal misfit at the implant-abutment interface. Scanning electron microscopes (SEM) were used to geometrically measure the marginal vertical discrepancy of a total of 80 specimens, classified into eight categories, according to the implant system and abutment. The data were statistically analyzed by Student’s paired t test, one-way and two-way ANOVA with the Bonferroni-Holm correction at the significance level of *p* = 0.05. Milled titanium abutments demonstrated the lowest misfit values in the implant systems analyzed. The marginal fit of all the groups was within the clinically acceptable range for implant prostheses.

## 1. Introduction

Dental implant rehabilitation, with over 40 years of scientific evidence, is a restorative alternative to the replacement of missing teeth widely used in clinical practice, due to the phycological and functional benefits of the patients, with high success and survival rates [1,2,3]. To provide a passive fit between the implant and its abutment, vertical misfit has been reported as an important requisite to ensure the aesthetic and functional long-term treatment success [4,5].

Passive fit is defined as a condition in which, in the absence of external loads, the prosthetic structure does not induce any tension on the implant and its components and thus does not induce any tension in the surrounding bone [6]. The misfit at the implant-abutment interface (IAI) can produce mechanical (screw loosening or fracture, abutment fracture) and biological complications (bacterial colonization, crestal bone loss and even loss of osseointegration) [1,5,7,8,9]. There is no consensus in the scientific literature about the clinically acceptable marginal gap. The average micro gap of the implant-abutment interface has been reported to be from 1 to 49 μm [10,11], while the average size of bacteria is 0.2–1.5 μm in width and 1–10 μm in length depending on the shape [12,13]. Jemt [14], who defined passive fit as the level of misfit without long-term clinical complications, considered that acceptable values of marginal misfit were within the 150 μm range.

Since the introduction of the Brånemark system in the 1960s and 1970s, a large number of implant systems have been developed [1]. Because the abutment sits on the external hexagon connection feature, this design has a high center of rotation relative to the implant and the screw is the only component securing the abutment. This type of connection has been associated with a certain amount of peri-implant bone loss, especially during the first year of performance [15,16]. Internal implant–abutment connections have been introduced to eliminate the mechanical complications associated with external connection and provide better aesthetics, long-term stability of implant-abutment complex and less crestal bone loss in the short–medium term as compared to external connections. The incidence of abutment screw loose or fracture is lower in internal implant connection [16,17,18,19]. The implant–abutment interface position at bone crest level or below may determine greater peri-implant bone remodeling. The platform switching determines a significant reduction of bone resorption reducing the bacterial colonization of the micro gap. Furthermore, the abutment height influences the marginal bone remodeling showing short abutments with greater marginal bone loss than long abutments [20,21].

For an implant restoration it is possible to choose between three types of abutments: stock (not customizable) commercial abutment; partially customized by milling but standard machined connection; and complete customized by casting or by using computer-aided design- computer-aided manufacturing (CAD-CAM) technology (milled or sintered) [4]. Laser sintering technology is a type of additive manufacturing that involves several advantages over the casting and CAD-CAM milling techniques: the ability to simultaneously manufacture multiple restorations, saving of the raw material, or requirement for few tools. This technique also reduces manufacturing time and costs [22,23,24].

A previous study reported that ceramic veneering procedures affect the discrepancy at the implant–prosthesis interface of metal frameworks fabricated by milling or additive manufacturing techniques [25]. In addition, ceramic veneer fracture is a frequent complication in implant-borne fixed restorations. Suspecting a hypothetical correlation between bruxism and ceramic veneer fractures, studies have been carried out, demonstrating a higher incidence of fractures in bruxers [26]. Bruxism is a common health problem that affects all age groups, social classes and cultures; with a prevalence ranging from 22.1 to 31% for awake bruxism and 9.7 to 15.9% for sleep bruxism [27,28]. Atsü et al. reported that the fracture strengths of Ti abutments were significantly higher compared to zirconia and ceramic-reinforced polyetheretherketone RPEEK abutments [29]. Likewise, previous studies reported that the fracture resistance of cemented all-ceramic crowns was significantly affected by the abutment material with the highest fracture resistance in metal abutments compared to zirconia abutments [30,31].

Titanium (Ti), owing to its well-documented biomechanical properties, is accepted as standard material for implant abutments [32,33]. Cobalt-chromium (Co-Cr) alloys have been widely used in dentistry for removable and fixed dental prostheses, mainly because they are strong, resistant to corrosion and relatively inexpensive, when compared to titanium, gold alloys and all-ceramic materials [34]. However, there are few studies that evaluate Co-Cr alloys for implant restorations.

The purpose of the present study was to evaluate the effect of two materials and methods of manufacturing implant abutments, CAD-CAM milled Ti and Co-Cr 3D laser sintered, on the marginal accuracy at the IAI on internal connection in four implant systems. The null hypothesis was that the abutment material or fabrication methods have no effect on the marginal discrepancies.

## 2. Materials and Methods

### 2.1. Preparation of Specimens

Eighty standardized machined methyl methacrylate (MMA) bases, whose dimensions (width × depth × height) were designed with AutoCAD 2011 (Autodesk, San Rafael, CA, USA), were manufactured using the EMCO Turn 342 numerical control lathe (EMCO Group, Hallein, Austria), which is governed by SINUMERIK (Siemens AG, Munich, Germany). All processing occurred in the Mechanical Workshop of the Physical Science Faculty (University Complutense of Madrid, Madrid, Spain). The dies were randomly assigned to one of the eight groups (n = 10 each according to the results of power analysis) and the groups were categorized according to the implant system (first letter: A, Avinent; G, GT-Medical; M, Mozo-Grau; P, Phibo) and the abutment material and manufacture technique (second letter: M, milled titanium; S, laser sintered Co-Cr): group AM, group AS, group GM, group GS, group MM, group MS, group PM and group PS (Table 1).

The next step was to insert the implants into the MMA bases. A central point of reference was marked to place the implants in the same vertical axis. The perforation was carried out in the prosthetic technology laboratory (Faculty of Odontology, University Complutense of Madrid, Madrid, Spain) with a parallelometer (PFG 100; Cendres and Metaux SA, Biel-Bienne, Switzerland) following the drilling standard protocol of each of the implant systems for the selected diameters. A magnetized structure was developed to contain the specimen in order to prevent its displacement during the drilling sequence.

A stock titanium commercial (not customizable) abutment was selected from each of the implant systems, for the milled abutments groups, with the following characteristics: straight abutment for cement and titanium grade V. All the milled Ti abutments were randomly screwed into their respective implant system with the specific screwdriver. The torque load was applied with the corresponding torque wrench following the manufacturer’s specifications, using the recommended torque for each of the standard abutment simulating the clinical procedure.

For the fabrication of the laser sintered Co-Cr abutment, four types of scan bodies were selected according to the implant system (Table 1). The scan body was screwed into the implant and was scanned using SMART desktop scanner (Open Technologies, FARO Europe GmbH and Co.KG., Korntal-Münchinengen, Germany). The sizes and designs were captured using Exocad software (Exocad GmbH, Darmstadt, Germany). The CAD data of abutments was sent to the direct metal laser sintering (DMLS) unit (SLM 125; SLM Solutions, Lübeck, Germany). The Starbond CoS Powder 45 (S&S Schefner GmbH, Mainz, Germany) was used with the following chemical composition as a percentage (wt %): 59% Co, 25% Cr, 9.5% W, 3.5% Mo and 1% Si. The grain size of the alloy powder was +10/45 μm. The layer thickness was fixed at 30 μm. The abutments were fabricated at the same time and sintered for six hours. The forty Co-Cr sintered abutments were screwed following the manufacturer’s torque load recommendations.

### 2.2. Fit Evaluation and Statistical Analysis

The marginal accuracy at the IAI was evaluated in the ICTS National Electron Microscopy Centre (University Complutense of Madrid, Madrid, Spain), by measuring the vertical marginal gap between the abutment margin and the cavo surface angle of the implant platform under a scanning electron microscope (SEM) (JSM 6400; JEOL, Tokyo, Japan). Before the SEM evaluation, in order to avoid electron beam distortion, the surface of the specimens were coated with 24 kt, 19.32 g/m^3^ density gold by a metallizer (Q15RS; Quorum Technologies, Sussex, UK) in vacuum under argon atmosphere. Two cycles of 7 min were performed: one for the buccal side and the other for the lingual one.

**Figure 1:**
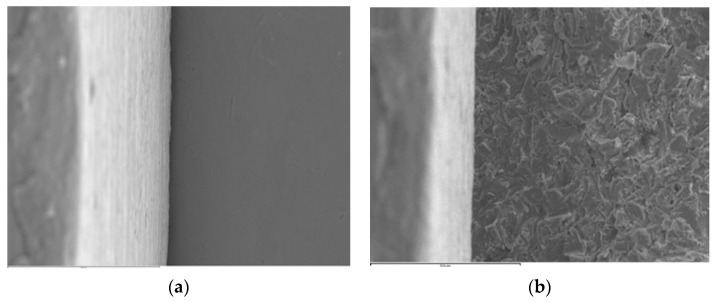
SEM images (1000×): (**a**) AM 4 V specimen, (**b**) AS 2 V specimen.

The JEOL 6400 SEM procedures increase in magnification of 15 to 30.000×, with 3.5 nm of resolution and a variable voltage of 0.5 to 40 kV. The selected working parameters for the samples were at 1000× magnification and 20 kV. Image acquisition was completed using the Link Pentafet energy dispersal detector (Oxford Instruments, Abingdom, UK). The images were transferred to a personal computer (Hewlett-Packard, Palo Alto, CA, USA) with specific software (INCA Suite 4.04; Oxford Instruments, Abingdon, UK) that digitalized the images after capturing them. Figure 1 shows two SEM images with non-measurable gaps; 1a corresponds to a milled Ti abutment and 1b to a Co-Cr laser sintered abutment. Both come from the same implant system (A). Prior to positioning the specimens on the sample holder of the microscope, and to ensure their correct location under the electron beam, a point in the middle of the buccal and lingual surfaces was marked with an indelible pen (Lumocolor permanent; Staedler Mars GmbH, Nurnberg, Germany). The images were coded with a number that indicates the number of the specimen within the group to which it belongs, followed by a letter V or L, indicating the location of the measurement: buccal (V) or lingual (L) surface.

**Figure 2:**
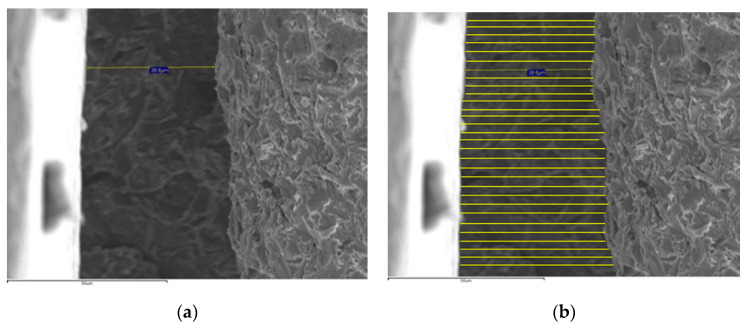
SEM images (1000×): (**a**) PS 10 L specimen, (**b**) Detail of the edited (**a**) image increasing the number of measurements.

The “calipper” tool of the SEM software only allows a calibrated measurement that measures the misfit in microns (μm). For this reason, the images were edited using Microsoft^®^ Paint 2013 (Microsoft Co., Redmond, WA, USA) to increase the number of measurements per sample. Twenty-nine parallel lines to the original INCA measurement, were added to obtain 30 measurements in buccal and lingual surfaces: a total of 60 measurements per specimen. The 60 measurements per specimen, as shown in Figure 2, were measured on a scale of 1:300 (Faber Castell, Stein, Germany). All the data were entered into a Microsoft^®^ Excel 2013 spreadsheet (Microsoft Corp, Redmond, WA, USA).

The statistical analysis was performed with the SPSS 22.0 software (SPSS Inc, Chicago, IL, USA). The normality of data was tested using the Shapiro-Wilk test. Student’s paired t test was performed to compare abutments type; one-way analysis of variance (ANOVA) was used to assess the influence of the implant system on the micro gap with the Games-Howell post hoc test, and two-way ANOVA was employed to compare the interaction of abutment and implant system. The level of significance was set at α = 0.05.

## 3. Results

The overall mean marginal misfit for the milled Ti abutments, regardless of the implant system, was 0.75 ± 1.27 μm, and the mean value for the laser sintered abutments was 11.83 ± 13.21 μm.

Table 2 shows the mean and standard deviation (SD) values for the experimental groups. The AM and MM groups demonstrated no mensurable misfit values. Likewise, both groups showed the lowest misfit for the laser sintered abutments.

The paired *t* test revealed that marginal misfit, regardless of the implant system, was significantly different among the abutments (*p* = 0.000) (Table 3). The misfit values for milled Ti abutments were significantly lower than those for sintered ones.

When analyzing the marginal misfit among the implant systems, regardless of the abutment, one-way ANOVA revealed significant differences (F = 4.807; *p* = 0.004). The post hoc test indicated differences between P and M groups (*p* = 0.028), showing the M group had the lowest discrepancies.

Two-way ANOVA revealed interaction between the abutment type and implant system (F = 7.683; *p* = 0.000) (Table 4). The study of the simple effects of the four implant systems was carried out through each of the levels of the abutment variable, showing that the differences in marginal misfit were only observed for the sintered abutments (F = 16.22; *p* = 0.000).

In the pairwise comparison (of the Two-way ANOVA) significant differences were found between the group PS with AS (*p* = 0.000), with GS (*p* = 0.001) and with MS (*p* = 0.000) groups (Table 5).

**Figure 3:**
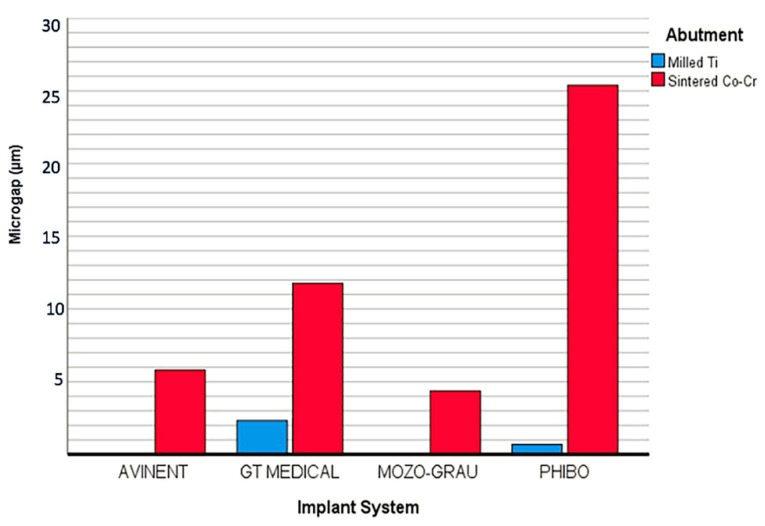
Graph of misfit values between the implant systems.

**Figure 4:**
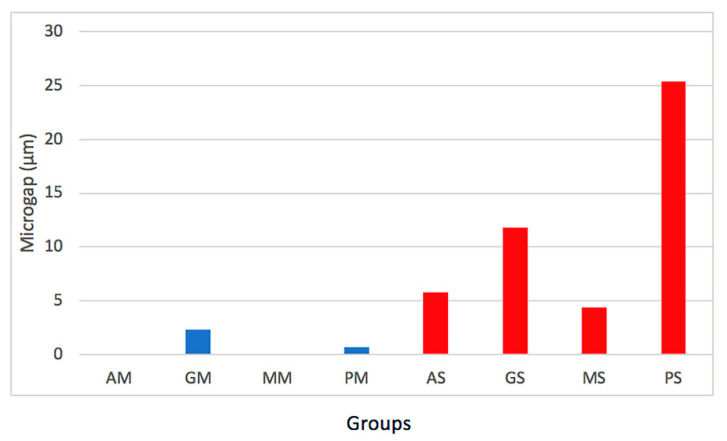
Graph of misfit values among the groups.

Finally, the simple effects were compared with each other to eliminate the influence exerted by the main effect of the factors on each of them. The effect of the type of abutment is not the same for the groups A and M compared to P group. No differences were observed between A and M groups for both type of abutments. Figure 3 showed the marginal misfit values between implant systems. Figure 4 exhibited the micro gap values for all the groups analyzed.

## 4. Discussion

This study evaluated the vertical marginal misfit at the IAI in internal hexagonal connection of four implants systems, with two types of abutments (original stock milled Ti and non-original custom CAD-CAM Co-Cr 3D laser sintered). The results obtained supported rejection of the null hypothesis, because significant differences were observed for the groups.

It has been stated that discrepancies and gaps between components are inevitable when two different pieces are placed together [35]. Optimal marginal accuracy is a crucial factor to maintain biological and mechanical equilibrium and decrease loading on the abutment, screw and supporting bone [36,37], factors related to the long-term success of implant restorations. Another clinical factor to be considered is the prosthetic abutment height. Spinato et al. demonstrated that internal hex platform-switched implants placed equicrestally and restored with short abutments present greater marginal bone loss than long abutments [20,21]. It is generally considered acceptable a micro gap of less than 10 μm [12,13,38]. The marginal misfit of all groups, except GS and PS, was within the clinically acceptable range of misfit.

No general guidelines exist on how to perform gap measurement restorations in vitro, or in vivo. This is one of the main reasons for variation reported among investigators. In most studies, statistical results are difficult to interpret because of variations in the sample size, measurements per specimen and measurement method used. The direct method is the most used. Some authors measured the IAI in non-sectioned specimens [18,35,39,40] and others prefer to measure in sectioned specimens [4,5,9,36]. The variations in the outcomes observed in screw-retained CAD-CAM restorations, depend on the technique used to measurement the marginal fit: SEM, stereomicroscope, optic microscope, scanning laser microscope or photogrammetry [9,17,18,35,39,41,42,43,44]. The shortcomings of a technique must be considered when interpreting results [45].

In this study, we evaluated the vertical marginal gap as the vertical marginal discrepancy measured from the abutment margin and the cavo surface angle of the implant platform, according to Holmes’ adapted classification [46] by direct viewing on an SEM to obtain external measurements. This technique has the advantage of being non-destructive. To standardize measurements, the specimens were examined under the same high power magnification and were placed in the base of the microscope to avoid movements, a crucial factor for the accuracy of this method. Moreover, this technique reduces the chance of error accumulation due to the preparation of a replica [41,47].

Another factor that varies among researchers is the number of measurements per specimen. It is considered that 50 measurements per specimen would allow a consistent estimation of the misfit [48,49]. To compensate smaller sample some authors have elevated the number of measurements from 50 to 100 sites [34,37,41,47].

There is not enough scientific literature about the use of Co-Cr alloys in fixed implant restorations. A few studies [24,36], have evaluated 3-unit implant-supported, screw-retained Co-Cr frameworks fabricated with different manufacturing techniques (conventional casting, CAD-CAM milling and selective laser melting). Selective laser melting technique showed lower marginal discrepancy (25 ± 14 μm) than conventional cast (35 μm) and CAD-CAM milling (68 μm) reported by Akçin et al. [24]. However, de Franca [36] observed lower vertical misfit values for conventionally fabricated frameworks with premachined abutments (11.8 μm) and for conventionally fabricated frameworks with castable abutments (12.9 μm).

Kim et al. [34] analyzed the marginal accuracy of Co-Cr crowns-copings fabricated by casting, CAD-CAM milling and 3D laser sintering, from an implant stock titanium abutment. The results reported that the 3D laser sintered group showed the highest vertical marginal discrepancies (72.5 μm), followed by CAD-CAM milled (51.5 μm) and casting technique (38.2 μm).

Regarding the use the of Co-Cr alloy abutments for single or multiple implant restorations, the available scientific research is sparse. However, the precision of fit of titanium and zirconia abutments has been widely studied, showing stock titanium abutments with lower discrepancies compared to zirconia abutments [1,4,5,9,18,37,50,51]. Differences in marginal misfit observed between the different authors may be due to the type of connection and the abutment material employed.

To the best of our knowledge, only two previous studies analyzed the misfit at the implant-abutment interface in 3D laser-sintered Co-Cr abutments. In both, a single implant system was evaluated, one with an external connection and the other one with internal connection. Fernandez et al. [51], reported a mean value of 0.73 μm for CAD-CAM milling Co-Cr abutments; 9.09 μm for casting group and 11.30 μm for 3D-laser sintered abutments. The results were similar to those obtained in the present study. Alonso et al. [40], reported no mensurable gaps for original stock abutments, consistent with the results obtained in the present study for AM and MM groups. However, the mean marginal vertical discrepancy values observed for laser sintered Co-Cr abutments was lower (2.5 μm) than those obtained in the study.

The common defects of laser sintering, reported by some studies, include porosity, distortion and delamination [52,53]. In the present study, differences in surface roughness were observed between milled Ti and Co-Cr 3D laser sintered abutments, indicating that the manufacturing technique is also a variable that influences the presence of micro gap. Other studies have also revealed that the rough mating surface produced by the laser sintered method, inevitably produces a micro gap between implant and abutment and hinders the achievement of a passive fit [51,54].

There were some limitations of this study. Only internal hexagonal implant connection was evaluated, and it would be interesting to compare the results with other implant connections.

Further research is needed to evaluate if the results found in the present study have clinical relevance, regarding the use of laser sintered Co-Cr abutment that would reduce the cost of fixed implant restorations through large-scale production at one time.

## 5. Conclusions

Milled titanium abutments demonstrated the lowest misfit values regardless of the implant system and within each implant system analyzed. The implant system and the type of abutment influenced the misfit. Laser sintered Co-Cr abutments misfit values are within clinically acceptable discrepancies. This technology can reduce the cost of an implant oral rehabilitation, allowing people with limited economic resources to have access to this type of treatment. Therefore, they could be an alternative to stock milled Ti abutments.

## Figures and Tables

**Table 1:** Characteristics of the implants, abutments and scan bodies used.

Test Group	Implants	Implant Material	Connection Design	Implant Diameter × Length (mm)	Platform (mm)	Abutments	Manufacturer
AM	Ocean IC STD	Ti grade V ELI	Internal hexagon	4.5 × 11.5 (Ref.1592)	4.1	Aesthetic abutment 4.5 × 1 mm 1724	Avinent Implant System SLU, Santpedor, Spain.
AS	Ocean IC STD	Ti grade V ELI	Internal hexagon	4.5 × 11.5 (Ref.1592)	4.1	Scan abutment 2801	Avinent Implant System SLU, Santpedor, Spain.
GM	HXI Tapered II	Ti grade IV	Internal hexagon	4.5 × 12 (Ref.D11417)	4.1	Straight abutment 4.5 × 1.5 mm G100010	GT Medical SL, Madrid, Spain.
GS	HXI Tapered II	Ti grade IV	Internal hexagon	4.5 × 12 (Ref.D11417)	4.1	Scan Body E004409	GT Medical SL, Madrid, Spain.
MM	InHex STD	Ti grade IV CP	Internal hexagon Internal morse connection	3.75 × 11.5 (Ref.23203711)	2.8	STD Inhex prepable 4 × 1 mm 23207010	Mozo Grau SA, Valladolid, Spain.
MS	InHex STD	Ti grade IV CP	Internal hexagon Internal morse connection	3.75 × 11.5 (Ref.23203711)	2.8	Scanbody Inhex STD c/t 41236002	Mozo Grau SA, Valladolid, Spain.
PM	TSADV S4	Ti grade IV	Internal hexagon	4.2 × 11.5 (Ref.04.115)	4.7	TSA S4 3.0 mm Abutment post 038.4030	Phibo Group, Senmenant, Spain.
PS	TSADV S4	Ti grade IV	Internal hexagon	4.2 × 11.5 (Ref.04.115)	4.7	Scanbody 002-TSA34	Phibo Group, Senmenant, Spain.

**Table 2:** Means and standard deviations (SD) misfit values (micrometers) for implant system and abutments material (M: milled Ti abutment; S: laser sintered Co-Cr abutment).

Test Group		n	Mean	SD
AVINENT (A) Santpedor, Spain	AM	10	0	0
	AS	10	5.81	10.29
GT MEDICAL (G) Madrid, Spain.	GM	10	2.32	1.70
	GS	10	11.77	12.41
MOZO GRAU (M) Valladolid, Spain.	MM	10	0	0
	MS	10	4.36	6.29
PHIBO (P) Senmenant, Spain.	PM	10	0.68	0.31
	PS	10	25.38	12.27

**Table 3:** Student’s paired *t* test.

x		Levene’s Test for Equality of Variances		*t*-Test for Equality of Means					95% Confidence Interval of the Difference	
		F	Sig.	f	df	Sig. (2-Tailed)	Mean Difference	Std. Error Difference	Lower	Upper
Marginal misfit	Equal variances assumed	92.32	0.000	5.278	78	0.000	11.08050	2.0992937	6.9011273	15.2598727
	Equal variances not assumed			5.278	39.724	0.000	11.08050	2.0992937	6.8367501	15.3242499

**Table 4:** Two-way ANOVA results for marginal discrepancy.

Source	Type III Sum of Squares	df	Mean Square	F	Sig.
Corrected Model	5249.839 ^a^	7	749.977	13.233	0.000
Intercept	3166.638	1	3166.638	55.872	0.000
Abutment	2455.550	1	2455.550	43.326	0.000
Implant System	1487.988	3	495.996	8.751	0.000
Abutment-I mplant System	1306.302	3	435.434	7.683	0.000
Error	4040.683	72	56.676	–	–
Total	12,497.160	80	–	–	–
Corrected Total	9330.523	79	–	–	–

^a^ R Squared = 0.563 (Adjusted R Squared = 0.520).

**Table 5:** Pairwise comparisons.

Dependent Variable: Marginal Discrepancy						95% Confidence Interval for Difference ^b^	
Abutment	Implant System (I)	Implant System (J)	Mean Difference (I−J)	Std. Error	Sig.^b^	Lower Bound	Upper Bound
Milled Ti	A	G	−2.325	3.367	1.000	−11.459	6.809
		M	−3.553 × 10^−15^	3.367	1.000	−9.134	9.134
		P	−0.680	3.367	1.000	−9.814	8.454
	G	A	2.325	3.367	1.000	−6.809	11.459
		G	2.325	3.367	1.000	−6.809	11.459
		M	1.645	3.367	1.000	−7.489	10.779
	M	A	−3.553 × 10^−15^	3.367	1.000	−9.134	9.134
		G	−2.325	3.367	1.000	−11.459	6.809
		P	−0.680	3.367	1.000	−9.814	8.454
	P	A	0.680	3.367	1.000	−8.454	9.814
		G	−1.645	3.367	1.000	−10.779	7.489
		M	0.680	3.367	1.000	−8.454	9.814
Laser Sintered	A	G	−5.968	3.367	0.483	−15.102	3.166
		M	1.447	3.367	1.000	−7.687	10.581
		P	−19.578 *	3.367	0.000	−28.712	−10.444
	G	A	5.968	3.367	0.483	−3.166	15.102
		M	7.415	3.367	0.185	−1.719	16.549
		P	−13.610 *	3.367	0.001	−22.744	−4.476
	M	A	−1.447	3.367	1.000	−10.581	7.687
		G	−7.415	3.367	0.185	−16.549	1.719
		P	−21.025 *	3.367	0.000	−30.159	−11.891
	P	A	19.578 *	3.367	0.000	10.444	28.712
		G	13.610 *	3.367	0.001	4.476	22.744
		M	21.025 *	3.367	0.000	11.891	30.159

Based on estimated marginal means.*. The mean difference is significant at the 0.05 level. ^b^. Adjustment for multiple comparisons: Bonferroni.
